# Physicochemical Characteristics and Anti-Inflammatory Activities of Antrodan, a Novel Glycoprotein Isolated from *Antrodia cinnamomea* Mycelia

**DOI:** 10.3390/molecules19010022

**Published:** 2013-12-19

**Authors:** Chun-Hung Chiu, Chiung-Chi Peng, Yaw-Bee Ker, Chin-Chu Chen, Arwen Lee, Wan-Lin Chang, Charng-Cherng Chyau, Robert Y. Peng

**Affiliations:** 1 Research Institute of Biotechnology, Hungkuang University, 34 Chung-Chie Road, Shalu District, Taichung 433, Taiwan; 2 Graduate Institute of Clinical Medicine, College of Medicine, Taipei Medical University, 250 Wu-Hsing Street, Taipei 11031, Taiwan; 3 Department of Applied Food Technology, Hungkuang University, 34 Chung-Chie Road, Shalu District, Taichung 433, Taiwan; 4 Grape King Biotechnology Inc., Zhongli City, Taoyuan County 320, Taiwan

**Keywords:** *Antrodia cinnamomea*, mycelia, glycoprotein, antrodan, structure, anti-inflammatory activity

## Abstract

*Antrodia cinnamomea* (AC) is a unique fungus found inhabiting the rotten wood of *Cinnamomum kanehirai.* A submerged liquid culture of AC has been developed and its bioproducts have been used to meet the market demand for natural fruiting bodies. AC exhibits anti-inflammatory, antitumor, antioxidant, and immunomodulatory effects. Previously, we isolated polysaccharide AC-2 from AC mycelia by means of alkali extraction with subsequent acid precipitation and found it had a pronounced anti-inflammatory effect. In this study, a novel polysaccharide named “antrodan” was obtained by further purification of AC-2 using Sepharose CL-6B column chromatography. Antrodan exhibited a molecular weight of 442 kD and contained a particularly high content of uronic acid (152.6 ± 0.8 mg/g). The protein content was 71.0%, apparently, higher than the carbohydrate content (14.1%), and thus antrodan was characterized as a glycoprotein. Its total glucan content was 15.65%, in which β-glucan (14.20%) was prominently higher than α-glucan (1.45%). Its FTIR confirmed the presence of β-linkages between sugars, and intramolecular amide bonds between sugars and amino acids. Its ^1^H-NMR spectrum showed that antrodan was a complex union of α- and β-glucans, which had (1→4)-linked α-Glc*p* and (1→3)-linked β-Glc*p* linkages to the carbohydrate chains via asparagine linked to protein site. Biologically, antrodan was confirmed to be totally non-detrimental to RAW 264.7 cell line even at dose as high as 400 μg/mL. It showed potent suppressing effect on the lipopolysaccharide-induced inflammatory responses in RAW 264.7 cell line. Moreover, antrodan significantly reduced the nitrogen oxide production at doses as low as 18.75 μg/mL.

## 1. Introduction

*Antrodia cinnamomea* (AC, syn. *Antrodia camphorata*, *Taiwanofungus camphoratus* and *Ganoderma comphoratum*) is a novel basidiomycete growing indigenously and uniquely in Taiwan. Its habit is to grow inside the empty rotten trunks of *Cinnamomum kanehirai* Hay [1]. Due to over deforestation, a limited quantity of host plants and its slow growth rate, the process for mass production of this fungus using submerged liquid culture systems is an emerging technology [2].

Many bioactive components have been identified in AC, which include triterpenoids, polysaccharides, benzenoids, benzoquinone derivatives, succinic and maleic acid derivatives [3]. The fruiting bodies of AC are enriched with triterpenoids that possess numerous biological activities, including immune-enhancing responses [4], fatigue-recovering effects [5], hepatoprotective effects [6], antioxidant, anti-inflammation and anticancer properties [3,7]. However, the most abundant and useful bioproducts obtained from the submerged liquid culture of AC mycelia are supposed to be polysaccharides, which have been widely described to potentially act as immunomodulatory products [8]. AC polysaccharides have shown various bioactivities like suppresssing the inflammatory mediator [9,10], alleviating allergic asthma [11], anti-angiogenic effects [12], modulating the immune system [13], modulating LPS-induced gene expression [14] and inhibiting tumorigenesis [15]. Literature elsewhere has indicated that neutral polysaccharide of AC mycelia revealed promising hepatoprotective effects [16]. A similar product, the protein-bound polysaccharide K (PSK, Krestin) has been approved in Japan since 1989 for use in combined chemotherapy to prolong the survival of patients with gastric cancer, colorectal cancer, and small-cell lung carcinoma [17,18]. However, documented chemical structure and biological function of AC glycoproteins are still lacking. Considering the important and distinct health-improving bioactivity of protein-bound polysaccharides, the potential value of glycoprotein obtained from AC mycelia is worth a deeper study.

In the present work, a glycoprotein, named antrodan, was obtained from the AC-2 polysaccharides fraction by further purification. Its chemical structure was elucidated. Meanwhile, its anti-inflammatory bioactivity was investigated with the murine cell line RAW 264.7. To compare the bioactivity, the commercialized polysaccharide ‘Biobran’ (Daiwa Pharmaceutical Co., Tokyo, Japan), a well-known immune response modulator made from rice bran using shitake mushroom enzymes, was used as the positive control.

## 2. Results and Discussion

### 2.1. Characterization of Antrodan

**Figure 1:**
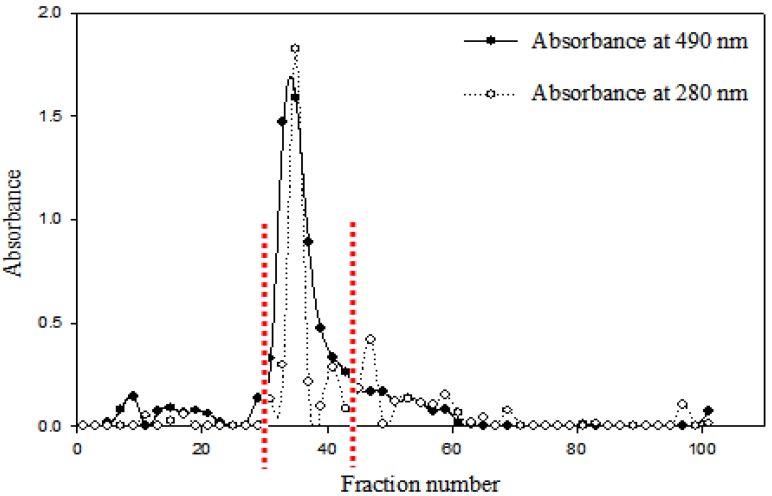
Gel filtration chromatogram of polysaccharides AC-2 prepared from AC mycelia using a Sepharose CL-6B column (3.0 × 82 cm)*.* Fractions (5.0 mL/fraction) were collected and assayed for the contents of sugars (at 490 nm) and proteins (at 280 nm). The vertical dashed lines indicated the fractions collected for preparation of antrodan.

**Figure 2:**
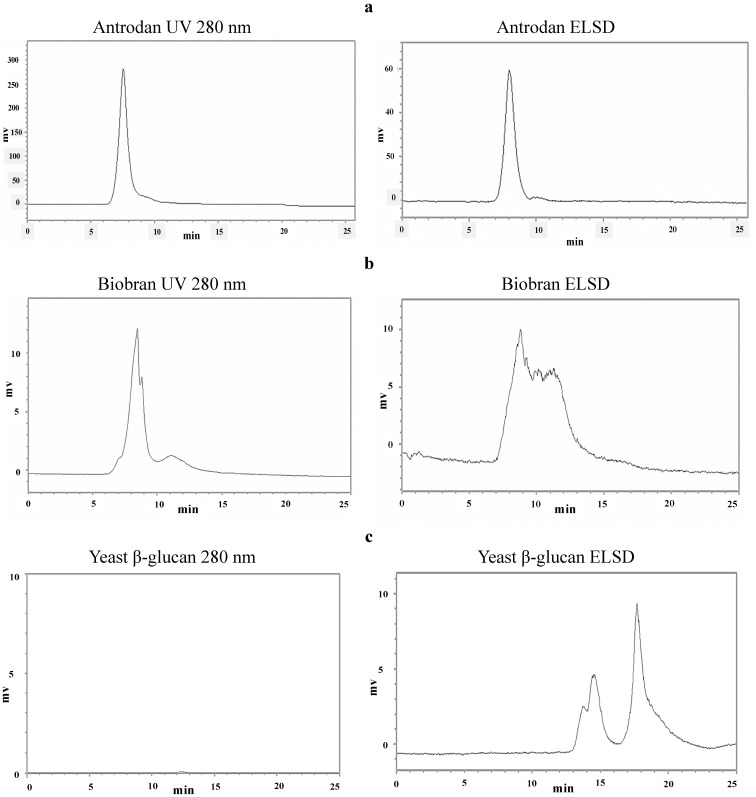
High performance size exclusion chromatograms of antrodan detected at UV 280 nm and ELSD absorption and the molecular weight estimation. The regression equation was obtained from the retention time *vs.* The MWs of pullunan reference set: 788, 404, 212, 112, 47.3, 22.8, 11.8, and 5.9 kDa. log Da = −0.466X + 10.009, R^2^ = 0.9937, where X is the retention time of the target polymer. Samples: antrodan, biobran, and yeast β-glucan.

The polysaccharide AC-2 fraction was prepared as previously reported [8]. For further purification, the AC-2 product was subjected to Sepharose CL-6B column chromatography, eluted with an isocratic ddw (pH 11.0 adjusted with NaOH) at a flow rate 0.5 mL/min. The obtained elution profile is shown in Figure 1. Fractions 29 to 43 were combined and dialyzed against distilled water. The concentrate was dried under a nitrogen-blow to give a purified polysaccharide named hereafter as “antrodan”. Alternatively, the concentrate was directly subjected to high performance size exclusion chromatographic (HP-SEC) analysis. As shown in Figure 2a, the absorption patterns obtained from the UV and the evaporative light-scattering detection (ELSD) revealed antrodan to be in a highly purified state. The average MW 442 kDa was obtained from the regression equation created by reference polymer compound ‘pullulan’:

log Da = −0.466X + 10.009, R^2^ = 0.9937 (1)

where X is the retention time of the target polymer.

As contrast, the prepared biobran presented at least three main components (Figure 2b). In biobran, the arabinoxylan with a xylose (in its main chain) and an arabinose polymer (in its side chain) have been identified as the major components [19]. Likewise, yeast β-glucan has been identified as a complex mixture of polysaccharides without any peptide moiety due to the lack of absorption at 280 nm (Figure 2c). The components of yeast β-glucan can be ascribed to β-1,6-glucosidic cross-linkings between β-1,3-glucan, mannan and chitin [20].

The original yield of crude polysaccharides AC-2 fraction was 10.38% [8]. Further purification yielded 9.19% of antrodan. Antrodan had a molecular weight 442 kD and a huge content of uronic acid (152.6 ± 0.0 mg/g). Its total glucan content reached 15.65%, in which β-glucan and α-glucan showed abundances of 14.20% and 1.45%, respectively (Table 1). For comparison, yeast β-glucan contained a total glucan 47.17% in which α-glucan and β-glucan represented 1.7% and 45.47%, respectively (Table 1).

**Table 1:** Comparison of average molecular weight, uronic acid and glucan contents among antrodan, biobran and yeast β-glucan.

Sample	Average MW (kDa) ^2^	Uronic acid (mg/g) ^3^	Glucan content (%)		
			α-glucan	β-glucan	Total
antrodan	442	152.6 ± 0.8	1.45	14.20	15.65
biobran	29	146.0 ± 1.0	ND ^4^	ND	ND
yeast β-glucan	~ ^1^	710.7 ± 2.3	1.70	45.47	47.17

^1^ Complex mixtures of polysaccharides as shown in Figure 2c of HPLC-ELSD chromatograms. ^2^ Average molecular weights of purified antrodan of *Antrodia cinnamomea* and biobran polysaccharide were determined by size exclusion chromatography. The calibration curve of polysaccharides MW established by using pullulan references ranging from 5.7 kDa to 788 kDa was used to analyze the prepared polysaccharide samples. ^3^ mg d-galaturonic acid /g extract weight. Each value was expressed as mean ± SD of triplicate samples. ^4^ ND: not detected.

**Figure 3:**
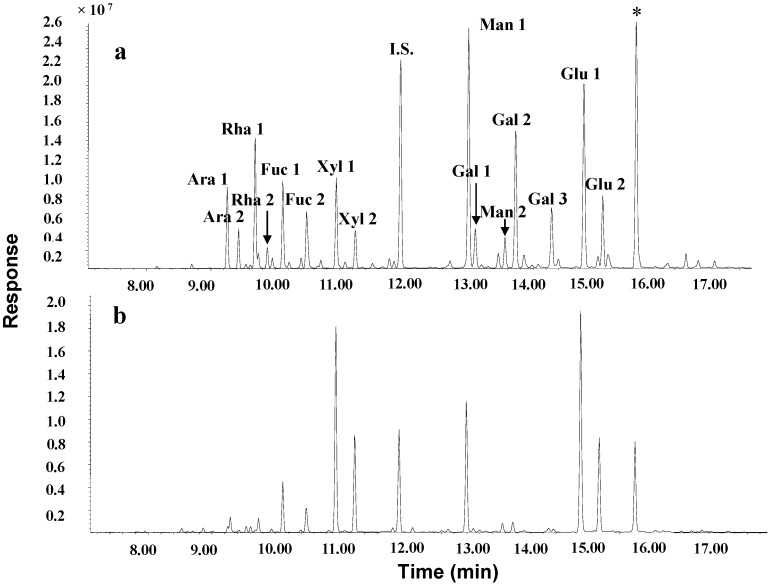
Total ion chromatograms of GC/MS analysis of monosaccharides. (**a**) authentic monosaccharides and (**b**) monosaccharides obtained from the hydrolyzed antrodan. Ara: arabinose, Rha: rhamnose, Fuc: fucose, Xyl: xylose, I.S.: internal standard arabitol, Man: mannose, Gal: galactose, Glc: glucose. *: Mannitol.

Antrodan contained in its polysaccharide moiety mainly four monosaccharides, *i.e.*, glucose (38.2%), xylose (33.7%), mannose (16.6%), and fucose (8.4%), respectively (Table 2) according to the GC/MS analysis (Figure 3), apparently indicating the characteristic composition of a xyloglucan. The peptidomoiety of antrodan contained 19 species of amino acids, mainly the essential amino acids (up to 53.66%), in which leucine (17.62%), valine (13.21%), isoleucine (10.53%), phenylalanine (9.85%) and alanine (9.12%) were the five major components (Table 3). The linkages between carbohydrate and protein in the complex biomolecules of newly isolated antrodan can occur in different glycopeptide bonds arranged in amino acids and sugars. *N*- and *O*-glycosylation occurring exclusively through GlcNAc-β-Asn and GalNAc-α-Ser/Thr linkages are the most well-known glycosylation modifications of proteins [21]. The comprehensive data in the present study revealed that the β-glycosylamine linkage of N-acetylglucosamine (GlcNAc) to asparagine (Asn) would be the most plausible form owing to the higher amounts of glucose (38.2%) linked to asparagine (2.23%) than those of galactose (1.80%) linked to serine (3.61%) or threonine (0.85%) (Table 2 and Table 3).

**Table 2:** The contents of carbohydrate, protein, and monosaccharide compositions of purified antrodan from *A. cinnamomea* mycelia.

Carbohydrate (%)	Protein (%)	Sugar component (%)						
		Arabinose	Rhamnose	Fucose	Xylose	Mannose	Galactose	Glucose
14.10	71.00	0.96	0.32	8.40	33.71	16.61	1.80	38.20

**Table 3:** Amino acid composition of glycoprotein antrodan.

Amino acid	% ^a^	Amino acid	% ^a^
Histidine ^b^	0.23	Asparagine	2.23
Isoleucine ^b^	10.53	Glutamic acid	6.60
Leucine ^b^	17.62	Glutamine	1.28
Lysine ^b^	1.24	Glycine	6.21
Methionine ^b^	0.13	Hydroxyproline	0.05
Phenylalanine ^b^	9.85	Ornithine ^c^	1.35
Threonine ^b^	0.85	Proline	4.40
Valine ^b^	13.21	Serine	3.61
Alanine	9.12	Tyrosine	4.70
Aspartic acid	6.73	-	-
Total			99.94

^a^ The results were analyzed from the bis *tert*-butyldimethylsilyl derivatives by using GC/MS. ^b^ Essential amino acids. ^c^ Ornithine was the hydrolyzed product from arginine during the acid hydrolysis.

**Figure 4:**
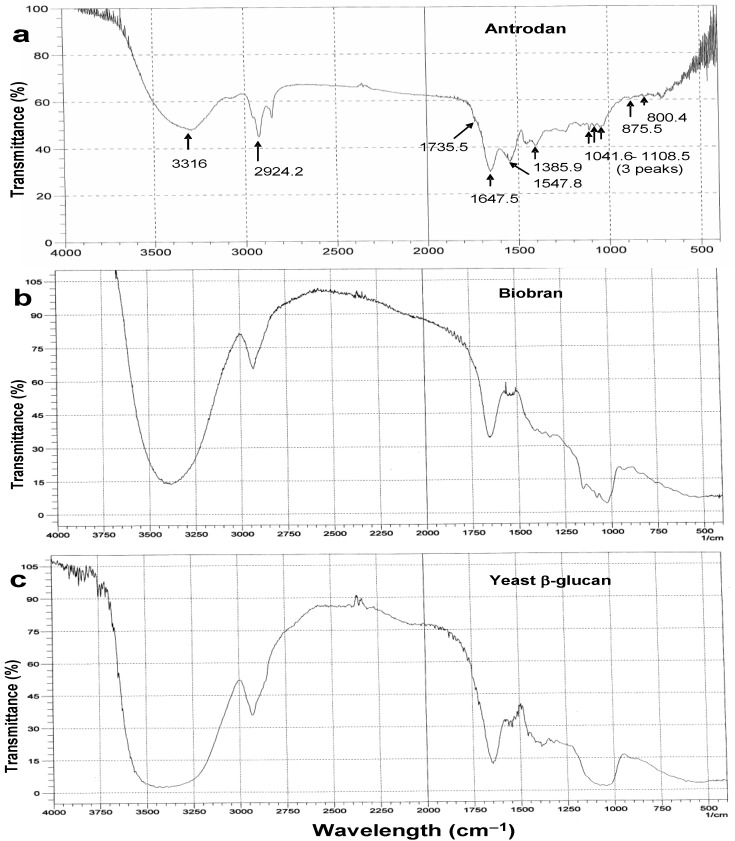
Comparison of the FTIR spectra. (**a**) antrodan, (**b**) biobran, and (**c**) yeast β-glucan. 2 mg of pure dry sample and 300 mg of pure dry KBr were mixed and pressed into a disc. The whole IR spectrum (400 to 4000 cm^−1^) was recorded on a FTIR spectrophotometer. Biobran is a reference of pure polysaccharide and yeast β-glucan acts as the β-glucan reference.

Figure 4a shows the IR absorption spectrum of antrodan. For comparison, Figure 4b and Figure 4c present the FTIR spectra of biobran and yeast β-glucan. Biobran (trade name BioBran/MGN-3), a potent candidate for treatment of patients with hepatic metastasis, is composed of denaturated hemicellulose from the rice bran hydrolyzed with multiple carbohydrate hydrolyzing enzymes of shiitake mushrooms [22].

The absorption peak assignments to antrodan were: 3316 cm^−1^, broad, s, ν_O-H_, (intermolecular hydrogen bonding; and amide A band N-H stretching, 3,300 H-bonded) (Figure 3a); 2924.2 cm^−1^, s, and 2890 cm^−1^, s, ν_CH2_; 1735.5 cm^−1^, s, ν_C=O_ (nonconjugated, may be uronic acid); 1647.5 cm^−1^, s, ν_C=O_ (amide I band, sec-amide, -CONH_2_); 1547.8 cm^−1^, s, δ_N-H_, or ν_C-N_ (amide II band, sec-amide, -CONH_2_, coupled C-N stretching and N-H bending); 1385.9 cm^−1^, s, δ_CH3_; 1250 cm^−1^, s, δ_C-N_; 1230–1000 cm^−1^ (the *sec*-cyclic alcohols of β-pyranoside). The three absorption peaks appearing within 1108.5–1041.6 cm^−1^, s, v_C-O_ (hint: 1275–800 cm^−1^ indicating the ν_C-__O__-C_ in monosugars like glucose or galactose; or changes in the endocyclic torsion angles of the furanose ring); 875.5 cm^−1^, m, (β-glycosidic linkage); 800.4 cm^−1^, s, ν_C-O-C_, m, (monosaccharide units). 800–640 cm^−1^, multiple absorption peaks, s, out of plane δ_N-H_. Taken together, these characteristics and IR absorption spectra indicated antrodan was a typical glucoxylan-protein complex.

**Figure 5:**
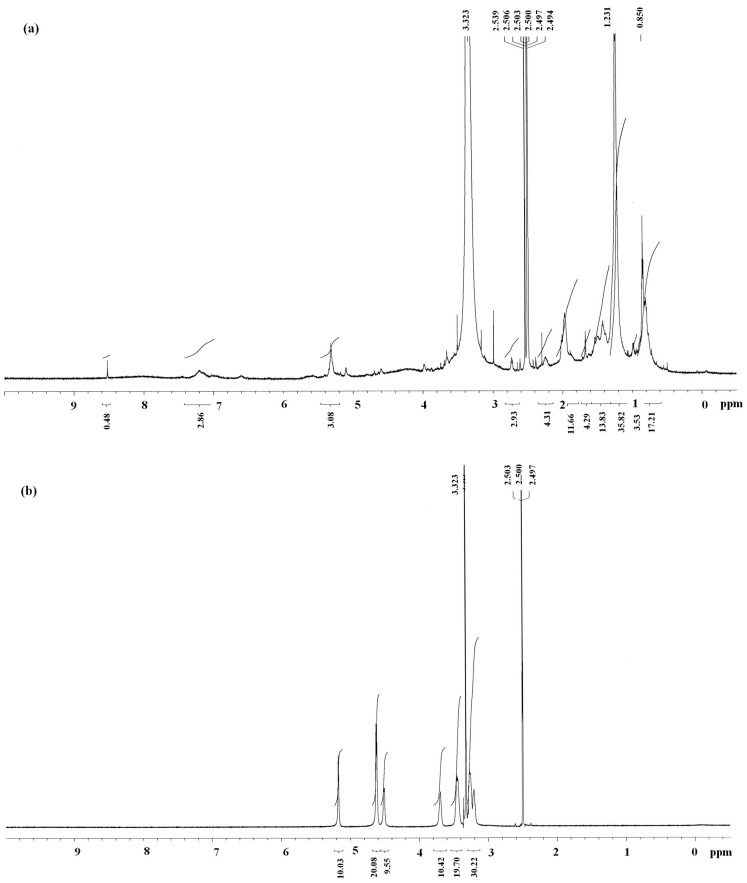
(**a**) ^1^H-NMR spectra of antrodan and (**b**) a standard β-d-(1→3, 1→6)-linked glucan of curdlan.

In order to determine the α- or β-configuration of glucose in the purified protein-bound polysaccharide antrodan, the ^1^H-NMR signals of antrodan (Figure 5a) were compared to those of curdlan (Figure 5b), a polysaccharide consisting of β-d-(1→3, 1→6)-linked glucose residues [23].

In the assignment of linkages in sugars, the ^1^H-NMR signals at 4.55 ppm assigned to the anomeric signal of (1→3)-β-d-glucan and 5.14 ppm for (1→4)-α-d-glucan [24] were demonstrated as α- and β-glucans complex in the polysaccharide part of antrodan (Figure 5a). In addition, the unique anomeric signals (4.4~5.5 ppm) and methyl (1.2 ppm) groups showed again the polysaccharide chemical structure of antrodan [25]. To distinguish the structural differences between polysaccharide and glycoprotein, we found that there was no polysaccharide signal appearing at 8.5 ppm in the downfield region, which is characteristic of the amide backbone of glycoproteins. Also, there was no signal appearing at 0.8 ppm (the strong methyl peak) in curdlan (Figure 5b). Apparently, signals at 8.5 ppm and the right side strong methyl peak at 0.8 ppm evidently indicate the random-coil protein conformation as a characteristic of antrodan [26].

### 2.2. Cell Viability Affected by Antrodan and LPS

**Figure 6:**
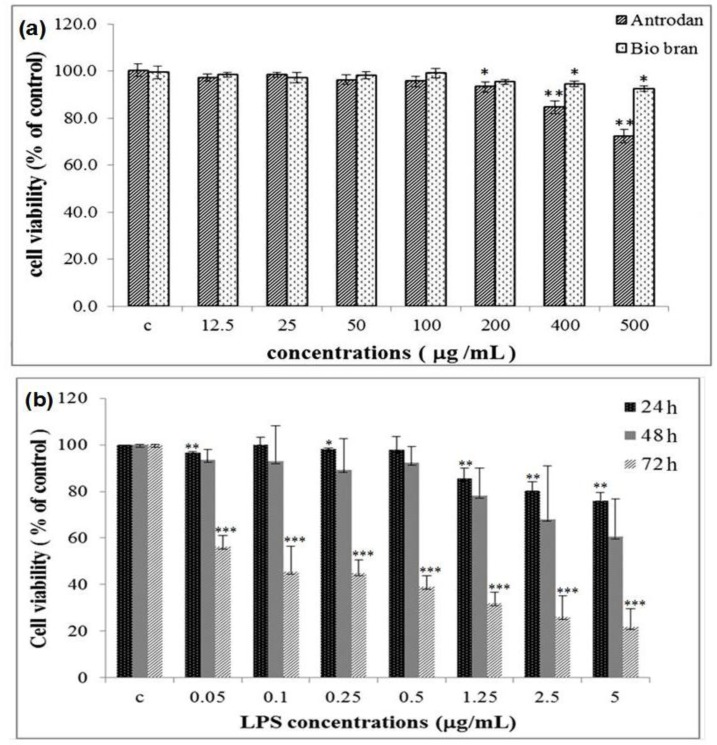
Effect of (**a**) antrodan and biobran, and (**b**) LPS on the cell viability of RAW 264.7 mouse macrophages. Cells were incubated with antrodan or biobran for 24 h (**a**) and incubated with lipopolysaccharide (LPS; 0.05~5 μg/mL) for 24, 48 and 72 h, respectively. Cell proliferation was evaluated by the MTT assay. Values are expressed as mean ± SEM of three independent experiments. * *p* < 0.05 *vs.* Control; ** *p* < 0.01; *** *p* < 0.001.

After 24 h of treatment of mouse macrophage RAW 264.7 cells with antrodan, cell numbers were counted individually. Figure 6a demonstrates that the viability of RAW 264.7 cells was not affected by antrodan, even at doses as high as 400 μg/mL (inhibition rate < 20%). Apparently, the purification procedures performed on the AC-2 polysaccharides has improved the cell toxicity of antrodan when compared to the previous study [8]. Apparent suppression (>20%) of cell viability occurred at dose ≥ 500 μg/mL (Figure 6a). However, the highest concentration of antrodan at 100 μg/mL was used to evaluate the anti-inflammatory effect elicited by LPS damage. At this concentration, the effects of antrodan on cell viability were 94.11% ± 4.36% and 91.48% ± 5.55% at 48 and 72 h, respectively (data not shown). LPS apparently inhibited the cell viability at 72 h in a dose responsive manner, with EC_50_ approximately at 0.08 μg/mL (Figure 6b). These results indicated that antrodan showed potential anti-inflammatory effects.

### 2.3. Antrodan Inhibited the *in vitro* LPS-Induced NO Production in RAW 264.7 Cell Line

**Figure 7:**
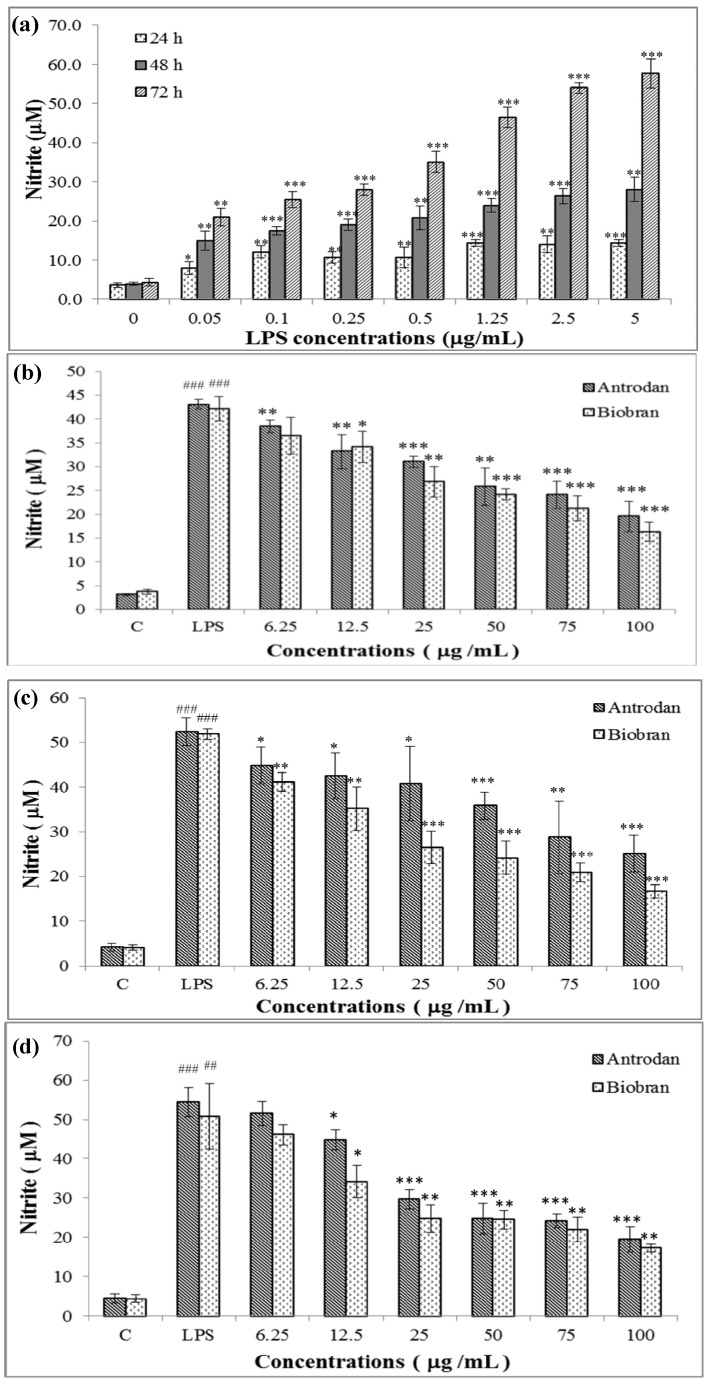
Effect of LPS on (**a**) NO production in RAW 264.7 mouse macrophages, (**b**) 24 h treatment, (**c**) 48 h treatment and (**d**) 72 h treatment with antrodan and biobran. Cells were treated with various concentrations of LPS for 24, 48 and 72 h, respectively. Nitrite content in the cultured medium was determined by a Griess reaction assay. Values are expressed as mean ± SEM of three independent experiments. The confidence levels * *p* < 0.05, ** *p* < 0.01, and *** *p* < 0.001 *vs.* control were determined by the Student *t*-test.

LPS induced the *in vitro* production of NO from the RAW 264.7 cell line in a dose- and time-responsive manner (Figure 7a). The NO production in the control was 4.70 ± 1.12 μM. LPS at 5 μg/mL induced the NO production to reach 56.11 ± 7.08 μM after 72 h, a 7.3-fold increase (*p* < 0.001) (Figure 7a). Antrodan was shown with moderately effective in suppressing the LPS-induced NO production (Figure 7b,c,d). The effectiveness of antrodan on the scavenging of NO production might be attributed to its high contents of uronic acid (Table 1). Previous studies have demonstrated that even among beta β(1→3) or β(1→6) glucans, slight differences in molecular weight, solubility, higher order structure like branching linkages and degree of branching, and attached proteins and lipids to backbones, higher order aggregates can result in great differences in innate immune activity [27].

In animal studies, after oral administration, the specific backbone 1→3 linear β-glycosidic chain of β-glucans cannot be digested. Most β-glucans enter the proximal small intestine and some are captured by the macrophages. They are internalized and fragmented within the cells, then transported by the macrophages to the marrow and endothelial reticular system. The small β-glucans fragments are eventually released by the macrophages and taken up by other immune cells leading to various immune responses [28]. Moreover, literature elsewhere indicated that β-glucans, *i.e.*, scleroglucan and laminarin, are able to be bound and internalized by intestinal epithelial cells and gut-associated lymphoid tissue cells and produced measurable plasma levels after oral administration of the glucans [29]. However, there has been no report about the bioactivity of antrodan yet. Study into the bioactivities and biological mechanisms underlying the anti-inflammatory effect of antrodan on LPS-induced acute liver injury is currently ongoing in the research group.

## 3. Experimental

### 3.1. Chemicals and Reagents

Trypan blue, sodium nitrite (NaNO_2_), 3-(4,5-dimethyl thiazol-2-yl)-2,5-diphenyltetrazolium bromide (MTT), sodium bicarbonate, lipopolysaccharide (LPS, *Escherichia coli* 055:B5), 2',7'-dichloro-fluoresein, N-(1-naphthyl)-ethylenediamine dihydrochloride, pyridine, acetic anhydride, sodium borodeuteride (NaBD_4_), sulfuric acid, phenol, carbazole, D-galaturonic acid, and tetramethoxypropane (TMP), and the authentic monosaccharides xylose, arabinose, mannitol, glucose, ribose, glucosamine, galacturonic acid, myo-inositol, lyxose, galactose, mannose, and rhamnose were provided by Sigma (St. Louis, MO, USA). Fetal Bovine Serum (FBS), L-glutamine solution (100 mM), penicillin-streptomycin (5000 units/mL and 5 mg/mL streptomycin) were purchased from Biological Industries (Beit Haemek, Israel). Dulbecco’s Modified Eagle Medium (DMEM) and trypsin-EDTA solution were provided by Hyclone (Logan, UT, USA). Protein assay kit was product of Bio-Rad (Hercules, CA, USA). Methanolic HCl (0.5 N), Sylon HTP kit, N,O-bis(trimethylsilyl)trifluoroacetamide (BSTFA) was product of Supelco (Bellefonte, PA, USA). Rat IL-6 ELISA kit was supplied by R&D (Minneapolis, MN, USA).

### 3.2. Isolation and Preparation of A. cinnamomea Polysaccharides

The freeze-dried mycelia were provided by the Biotechnology Center of Grape King Inc., Chung-Li City, Taiwan and voucher specimens of the materials were deposited at the laboratory of Biological Products of the Institute of Biotechnology, Hungkuang University, Taiwan. The production of mycelia and the polysaccharide fractionation were prepared as previously described [8]. In brief, the defatted mycelia powder (1 kg) obtained by supercritical fluid extraction was extracted with reflux three times with 20 L of double-distilled water (DDW) at 90 °C with constant stirring at 400 rpm for 2 h to remove water soluble matters including sugars and amino acids, *etc.* After the mixture was filtered, the residues were dried *in vacuo* and then were extracted with hot alkaline solution (pH 9.0, 1:10, w/v) at 80 °C for three times, 2 h each time. The extracts were filtered with aspiration after cooling. To the filtrates were added 1 N HCl to adjust the pH to 4.0, then concentrated and lyophilized to afford base-soluble extracts. The extracts were resolved in 10 volumes DDW and then a 3-fold volume of ethanol (95%) was added into the solution to precipitate the base-soluble polysaccharides, and then collected and lyophilized and the product was designated AC-2.

### 3.3. Isolation of Antrodan by Gel Permeation Chromatography

According to our previous report [8], to pulverized AC-2 (100 mg) was added NaOH solution (0.05 M, 10 mL) and heated to 50 °C while stirring to facilitate the dissolution. The solution was centrifuged at 13000 *×g* for 5 min to eliminate the insoluble residue. The supernatant containing antrodan was firstly eluted with a 0.05 M NaOH (containing 0.02% of NaN_3_) solution on a Sephadex G-100 column (2.5 × 100 cm) at a flow rate of 0.5 mL/min. Eluents were collected by a fraction collector (ISCO Retriever 500, Lincoln, NE, USA), each 6 mL in a tube. The collection was continued until a total of 100 tubes were reached. Eluents in tubes (16th to 40th) were combined and concentrated. The resulting concentrates were then loaded onto a Sepharose CL-6B column (3.0 × 82 cm) equilibrated with a ddw (pH 11.0 adjusted with NaOH) and then eluted with the same solution to separate polysaccharides at a flow rate 0.5 mL/min. The eluents were collected with a fraction collector (SF-2120, Advantec MFS, Inc., Dublin, CA, USA) at a velocity 1 tube/10 min to reach a total of 100 tubes. The content of total sugar in the eluent was determined (optical density at 490 nm), in parallel the optical density at 280 nm was monitored. Based on the optical density at 280 nm the main fractions (29th to 43rd tubes) were selected and combined. The combined solution was lyophilized (designated as antrodan). A serial physical chemical analysis was carried out. Biobran and yeast β-glucan were selected as the reference compounds to compare with antrodan.

### 3.4. Preparation of Biobran Polysaccharide

To the Biobran powder (Daiwa Pharmaceutical Co. Ltd., Tokyo, Japan) was added ddw in a ratio of 1:10 (w/v). The mixture was refluxed at 80 °C with constant stirring at 400 rpm. The extraction was continued for 2 h, and the solution was left to cool at ambient temperature and centrifuged at 6,000 *×g* for 10 min. The supernatant was filtered with Toyo No.1 filter paper. The filtrate was kept. The extraction was repeated for three times and the filtrates were combined. To the combined filtrate was added 3-fold ethanol (95%) to precipitate the polysaccharides. The solution was left to stand overnight at 4 °C and centrifuged at 6,000 *×g* for at 10 min. The precipitate was lyophilized to give biobran polysaccharide.

### 3.5. Physicochemcial Characterization of Antrodan

#### 3.5.1. Structural Analyses

The molecular weight of antrodan was determined by high performance size-exclusion chromatography (HP SEC) using the tandem columns of PolySep-GFC-P (75 × 7.8 mm) and PolySep-GPC-P 4000 (300 × 7.8 mm, Phenomenex, Torrance, CA. USA) installed with a pre-column PolySep-GFC-P (ℓ × id = 75 × 7.8 mm) and serially coupled to ultraviolet (UV, 280 nm) and evaporative light scattering detectors (ELSD, SEDERE, SA, Alfortville, Cedex, France). The ELSD *s*ystem was operated with the following settings: a drift tube temperature of 50 °C, a gain of 5, and a nebulizer nitrogen gas pressure of 2.3 bar. Deionized water was used as the mobile phase with the flow rate at 0.8 mL/min. The column oven temperature was 40 °C. The reference polymer used was pullulan having a serial different molecular weights: 788, 404, 212, 112, 47.3, 22.8, 11.8, 5.9 kDa. The regression equation shown below was used for estimation of the molecular weight of antrodan:

log Da = −0.466X + 10.009, R^2^ = 0.9937 (2)

where X is the retention time of the target polymer.

Infrared spectra were obtained on a Shimadzu FTIR 8400s Fourier Transform infrared spectrometer. Samples of antrodan, after completely desiccated, was thoroughly mixed with IR grade KBr (1:100), tabletted and fixed onto the sample chamber. The FTIR spectra were scanned within 4,000–400 cm^−1^ at a resolution of 4 cm^−1^. For each sample scans were repeated ten times to ensure the precision. ^1^H-NMR spectra were obtained on a Varian MR 400 MHz instrument. The samples were dissolved in deuterated dimethyl sulfoxide (DMSO-*d_6_*) at a concentration of 20 mg/0.6 mL.

#### 3.5.2. Determination of Total Sugar Content

According to Masuko *et al.* [30], the total sugar content was determined by the phenol-H_2_SO_4_ method. A calibration curve was established using authentic glucose as the reference compound, from which the amount of total sugar was estimated.

#### 3.5.3. Determination of Total Protein Content

The Bradford protein binding assay [31] using Coomassie Brilliant Blue G-250 dye was followed to determine the total protein content. A calibration curve was established using BSA as the reference compound, from which the total protein content was estimated.

#### 3.5.4. Determination of Monosaccharide Composition

To antrodan (1 mg) methanolic HCl (0.5 N, 200 μL) was added. After the mixture was left to react at 80 °C for 14–16 h, then MeOH (500 μL), pyridine (10 μL) and acetic anhydride (50 μL) were successively added. The mixture was vigorously agitated and centrifuged. The reaction mixture was left to stand for 15 min. After N_2_ gas blow dried, Sylon HTP kit (200 μL, HMDS/TMCS/pyridine = 3:1:9) was immediately added to avoid oxidation. After thorough agitation, the mixture was centrifuged and left to stand for 15–30 min. The volume of the solution was reduced under N_2_ gas blowing to 100 μL. To the mixture *n*-hexane was added, agitated and centrifuged for 5 min. The supernatant was transferred to a new tube. The *n*-hexane extraction was repeated three times. The three supernatant extracts were combined and dried under a N_2_ gas blow. The residue was stored at −20 °C for use. For GC/MS analysis, the residue was dissolved in methanol, an aliquot 1 μL was sampled and was analysed using an Agilent 6890 gas chromatograph (GC) coupled to an Agilent 5973A MSD mass spectrometer (EI mode, 70 eV, Agilent Technologies, Santa Clara, CA, USA) equipped with HP-5MS capillary column (ℓ = 30m, id = 0.25 mm, Agilent Technologies). The column temperature was held at 60°C for 1 min and then programmed to 150 °C at 25 °C/min, from 150 °C to 200 °C at 5 °C/min and finally from 200 °C to 300 °C at 10 °C/min. Temperatures for GC injector and GC–MSD interface were 250 and 265°C, respectively. The injection port and detector temperatures were set at 250 °C/265 °C. The carrier gas used was helium operated at a flow rate 1 mL/min. Authentic monosaccharides were treated similarly with the same protocol. The amount of each monosaccharide was estimated by comparing with the each corresponding authentic peak.

#### 3.5.5. Analysis of Amino Acid Composition

Method of Sobolevsky *et al.* [32], slightly modified in this laboratory, was adopted to conduct the amino acid analysis. This method has the advantages to successfully detect glutamine and asparagine. Briefly, the pulverized antrodan (2 mg) was placed in the microreactor (1 mL, Supelco, Bellefonte, PA, USA), heated at 60 °C to dryness under a N_2_ gas blow. HCl (6 N, 1 mL) was added and heated at 100 °C for 24 h. The reaction vessel was cooled at ambient temperature and evaporated at 40 °C under N_2_ gas blow. Methanol (0.2 mL) was added and evaporated. The process was repeated for three times until the moisture was completely driven off. To the residue, norleucine (100 μL containing 0.4 mg in pyridine to serve the internal reference standard) was added. The mixture was agitated to facilitate the dissolution. The derivatization reagent *N*-methyl-*N*-(*tert*-butyldimethylsilyl) trifluoroacetamide (MTBSTFA, 100 μL, containing 1% TCMS) dissolved in pyridine (100 μL) and acetonitrile (80 μL) were added, agitated and mixed well. The derivatization reaction was carried out at 120 °C for 30 min. One μL of the aliquot was subjected to GC/MS analysis. The authentic amino acid samples, each 0.3 mg, were similarly treated. The GC/MS chromatograph equipped with a capillary column HP-5MS (ℓ × id = 30 m × 0.25 mm, film thickness = 0.25 μm; Agilent Technologies) was used. The injection port was held at 280 °C. The flow rate of carrier gas helium was operated at 1.0 mL/min. The temperature of the GC and MS interface was held at 290 °C. The elution temperature was programmed from 40 °C to 280 °C at 10 °C/min, and held at which for 10 min. The split rate was set at 40:1, and measured with electron impact (EI) 70 eV. A full scan status was used to ensure the complete catching for all derivative fragments. The scan range was set between 40 and 700 *m/z*. MSD ChemStation Software (Agilent Technologies) was used for data collection and analysis.

#### 3.5.6. Determination of Uronic Acid Content

The method of Galambos [33] was followed to carry out the assay for uronic content. The optical density was read at 550 nm. The authentic D-galacturonic acid was similarly treated to establish the calibration curve. Based on the calibration curve the amount of uronic acid was calculated.

#### 3.5.7. Determination of Glucans

##### 3.5.7.1. Total Glucans

Total glucans was carried out by using the mushroom and yeast beta-glucan kit (Megazyme, Wicklow, Ireland) according to the manufacturer’s instruction. Briefly, antrodan powder (100 mg) was transferred into a spiral test tube and 1.5 mL HCl (37% v/v) was added and agitated to mix well. The mixture was heated at 30 °C for 45 min and agitated every 15 min. To the reaction mixture 10 mL water was added and agitated to mix well. The mixture was heated at 100 °C for 5 min after removing the cap. The tube was recapped and the heating was continued for 2 h. The finished reaction mixture was left to cool at ambient temperature. KOH solution (2 N, 10 mL) was added and the volume was made to 100 mL with 200 mM sodium acetate (pH 5.0). The solution was gently shaken to obtain homogenous solution and centrifuged at 1500 *×g* for 10 min to remove any contaminants. The supernatant (0.2 mL) was transferred to two spiral tubes, each 0.1 mL. To each tube 0.1 mL mixture of exo-1,3-β-glucanase (20 U/mL) and β-glucosidase (4 U/mL, in 200 mM sodium acetate, pH 5.0) were added, mixed well and kept at 40 °C for 20 min. To the reaction mixture 3 mL glucose oxidase (12 U/mL) with peroxidae 0.65 U/mL (GOPOD) was added and mixed well. The enzyme reaction mixture was left to react at 40 °C for 20 min. The mixture was left to cool in ambient temperature. The optical density was taken at 510 nm.

##### 3.5.7.2. α-Glucans

Antrodan powder (100 mg) was transferred into a spiral test tube. KOH (2 M, 2 mL) was added and stirred in ice bath with constant stirring for 20 min. To the solution 8 mL of sodium acetate (1.2 M, pH 3.8) was added and immediately followed by adding 0.2 mL aliquot of amyloglucosidase (1630 U/mL) + invertase (500 U/mL), and mixed well. The enzyme solution was left to react for 30 min at 40 °C while agitated frequently. The reaction mixture was centrifuged at 1500 *×g* for 10 min and the supernatant volume was made to 10.3 mL with sodium acetate solution (200 mM, pH 5.0). The supernatant (0.2 mL) was transferred to two spiral tubes, each 0.1 mL, and successively 0.1 mL sodium acetate solution (200 mM, pH 5.0) and 3 mL GPPOD reagent, mixed well. The solution was kept at 40 °C for 20 min, cooled to roomtemperature The optical density was read at 510 nm.

For calculation, the following equations were used:

Total glucan (% w/w) = ΔE × (F/W) × 90
(3)

α-glucan (% w/w) = ΔE × (F/W) × 9.27 (note the final volume was 10.3 mL)
(4)

β-glucans = total glucans − α-glucans
(5)

where ΔE is the OD_sample_ − OD_blank_, F = 100 × (W_ag_)/OD_ag_, W_ag_ is the weight of the authentic D-glucose used, OD_ag_ is the optical density of authentic d-glucose, W is the weight of sample.

### 3.6. Macrophage RAW 264.7 Cell Cultivation

To Dulbecco’s Modified Eagle’s Medium (DMEM, HyClone, Logan, UT, USA) 10% FBS and 1% penicillin (10,000 units/mL), streptomycin (10 mg/mL) and 2 mM L-glutamine were added. RAW 264.7 cell line was rinsed twice with 3 mL 0.01 M phosphate buffered saline (PBS) and 1 mL 0.25% (1×) trypsin-EDTA was added. The cells were incubated at 37 °C under 5% CO_2_ atmosphere in incubator.

### 3.7. Effect of Antrodan and Biobran on the Cell Viability

The method of Mosmann *et al.* [34] was followed to examine the cell viability. RAW 264.7 cell line was seeded onto 96-well plate at 6,000 cells/well. The next day, the cells were incubated with various concentrations of antrodan or biobran for 24, 48 or 72 h, and cell viability was determined by using a colorimetric MTT [3-(4,5-dimethylthioazol-2-yl)-5-(3-carboxymethoxyphenyl)-2-(4-sulfophenyl)-2*H*-tetrazolium] test. In brief, to the culture medium MTT (100 µL, 0.5 mg/mL) was added and left to react for 2 h. The culture medium was centrifuged at 12,000 *×g* and the supernatant was removed. DMSO (1 mL) was added to dissolve the formazan crystal. The optical density was read at 570 nm with an ELISA reader.

### 3.8. Effect of LPS on the Cell Viability

RAW264.7 cell line was seeded at 6,000 cells/mL to 96 well plate and incubated with DMEM for 24 h. LPS was added at concentration range 0.0–5.0 μg/mL as indicated. The incubation was continued for 72 h. The MTT assays were conducted at 24, 48 and 72 h after addition of LPS.

### 3.9. Effect of LPS and Antrodan on NO Production in Cell Line

The method of Green *et al.* [35] was adopted with slight modifications. Briefly, for determination of serum NO, VCl_3_ solution (100 μL, 0.8% in 1N HCl) was transferred onto a 96-well plate, then serum (100 μL), Griess reagent (100 μL, prepared by mixing 1:1 v/v 1% sulfanilamide in 5% phosphoric acid and 0.1% naphthylethylenediamine dihydrochloride in water) were added sequentially. The mixture was kept away from the direct sunlight and left to react for 15 min. The optical density was read with ELISA reader at 540 nm. A calibration curve was established by using authentic NaNO_2_, from which the nitrite and nitrate contents were estimated.

For determination of the NO production in cell line, RAW264.7 cell line was seeded at 6,000 cells/mL to 96 well plate and incubated with DMEM for 24 h. LPS was added at concentration range 0.0–5.0 μg/mL as indicated. The incubation was continued for 72 h. The culture medium (100 μL) was analyzed at 24, 38 and 72 h, respectively for NO production using the similar protocol mentioned in the above.

### 3.10. Statistical Analysis

Data obtained were statistically treated with One-Way Analysis of Variance (ANOVA) analysis. Tukey’s test or least significant difference test (LSD) was used to analyze the difference of significance. The *p* < 0.05 was considered to be significantly difference between groups.

## 4. Conclusions

Antrodan, a unique glycoprotein, has been isolated from AC mycelia. Antrodan has revealed promising *in vitro* antiinflammatory effects, suggesting antrodan could be a candidate for further development of functional foods.
